# No differences in patient‐reported outcomes (PROMs) and complications after anterior cruciate ligament (ACL) reconstruction with bone–patellar tendon–bone (BPTB) or hamstrings (HT) in patients aged 50 or older

**DOI:** 10.1002/jeo2.70371

**Published:** 2025-07-18

**Authors:** Rodrigo Olivieri, José I. Laso, Carlos Rojas, Nicolás Franulic, Felipe Bustos, Roberto Oyarce, Nicolás Gaggero, Alberto Grassi

**Affiliations:** ^1^ Knee Unit, Orthopaedic Department Hospital del Trabajador Santiago Chile; ^2^ Universidad Andrés Bello, Hospital del Trabajador, Facultad de Medicina Santiago Chile; ^3^ Knee Unit, Orthopaedic Department Hospital Militar de Santiago Santiago Chile; ^4^ Facultad de Medicina Universidad de Los Andes Santiago Chile; ^5^ Hospital del Trabajador Santiago Chile; ^6^ Clinica Ortopedica e Traumatologica II, IRCCS Istituto Ortopedico Rizzoli Bologna Italy

**Keywords:** anterior cruciate ligament, bone–patellar tendon–bone, hamstring, reconstruction, 50 years old and over

## Abstract

**Purpose:**

This study aimed to compare postoperative clinical outcomes, including patient‐reported outcome measures (PROMs), patellofemoral symptoms, and complications, in patients over 50 years old who underwent anterior cruciate ligament (ACL) reconstruction with either bone–patellar tendon–bone (BPTB) or hamstring tendon (HT) autografts. We hypothesised no significant differences in outcomes between the two techniques in this age group.

**Methods:**

A retrospective cohort study was conducted using institutional records of patients over 50 years old who underwent isolated ACL reconstruction (defined as ACL reconstruction without associated ligament injuries), with or without concomitant meniscal procedures, using BPTB or HT autografts between January 2016 and December 2022, with a minimum follow‐up of two years. Postoperative outcomes were assessed using PROMs, including the Lysholm score, Kujala score, and the Knee injury and Osteoarthritis Outcome Score Quality of Life subscale (KOOS QoL), complication rates, and the need for revision surgery. Statistical analyses included independent t‐tests, Mann–Whitney *U* tests, chi‐square tests and multivariate regression analyses.

**Results:**

A total of 83 patients met the inclusion criteria (45 HT and 38 BPTB). The mean age was 53.5 years (SD 3.0), with a mean follow‐up of 56.3 months. No significant differences were found in Lysholm (HT: 83.1, BPTB: 86.1; *p* = 0.934), Kujala (HT: 82.2, BPTB: 84.5; *p* = 0.901), or KOOS QoL scores (HT: 69.0, BPTB: 68.7; *p* = 0.649). The incidence of complications and the need for revision surgery were similar between groups.

**Conclusions:**

ACL reconstruction in patients over 50 years old using BPTB or HT autografts resulted in comparable clinical outcomes, PROMs, and complication rates. In this cohort, graft type did not appear to significantly influence postoperative results.

**Level of Evidence:**

Level III, retrospective cohort study.

**Abbreviations**
ACL: anterior cruciate ligament
AL: anterolateral
AM: anteromedial
BMI: body mass index
BPTB: bone–patellar tendon–bone
CI: confidence interval
HT: hamstring tendon
IKDC: International Knee Documentation Committee
KOOS: Knee Osteoarthritis Outcome Score
LEAP: lateral extra‐articular procedures
MRI: magnetic resonance imaging
MVA: motor vehicle accident
OR: odds ratio
PASS: patient acceptable symptom state
PROMs: patient‐reported outcome measures
QoL: quality of Life
ROM: range of motion
SD: standard deviation

## INTRODUCTION

The increasing life expectancy and demand for sports activities among individuals aged 50 years and older have led to a rise in anterior cruciate ligament (ACL) injuries and reconstructions in this population [5, 47]. Classically, these patients were managed conservatively [8, 25], however, the outcomes of conservative treatment may be suboptimal in some cases due to persistent rotational or anteroposterior instability, as well as associated meniscal and cartilage injuries [8, 50]. In this regard, although nonoperative management remains a valid option for selected patients in this age group [20, 44], recent studies have shown favourable outcomes of surgical treatment for ACL injuries in patients over 50 years old [12, 14, 16, 51, 59], even comparable to those in younger patients [10, 22, 37, 53].

The choice of graft type for ACL reconstruction remains controversial in the general population [3, 13, 18, 49, 55] and even more so in older patients, partly due to the limited available evidence [4]. In this context, while the bone–patellar tendon–bone (BPTB) graft has long been considered the gold standard for ACL reconstruction [35, 39, 55], current trends indicate that the hamstring tendon (HT) graft is the most commonly used by orthopaedic surgeons, followed by BPTB [7, 19, 56].

One of the commonly cited reasons for using HT in ACL reconstruction is the lower incidence of patellofemoral symptoms and anterior knee pain compared to BPTB [32, 40, 41, 54]. However, there is no complete consensus on this matter [6, 48].

In this regard, it is possible that donor site morbidity could be more pronounced in older patients or those with lower physiological reserve. Given that the use of allografts is not an available option in all centres, the choice of autograft type may play a more significant role in this group compared to younger patients.

The aim of this study was to evaluate the postoperative clinical outcomes, including patient‐reported outcome measures (PROMs), patellofemoral symptoms, and complications in patients over 50 years old with ACL rupture who underwent reconstruction using either HT or BPTB autografts. Our hypothesis was that there would be no significant differences in the evaluated postoperative outcomes between the use of HT and BPTB autografts in this age group.

## MATERIALS AND METHODS

### Ethics committee

The study was conducted in accordance with the ethical standards outlined in the Declaration of Helsinki and Resolution 008430 of 1993. It was approved by the Institutional Ethics Committee (internal code CEC/02/2025), and informed consent was obtained from all patients participating in the study at the time the functional scales were administered.

### Study design

The present study was conducted according to the STROBE guidelines. A retrospective cohort study was conducted by analysing records of patients with isolated ACL rupture, aged over 50 years, who underwent surgical treatment at a single centre. The institutional database of electronic medical records for patients who underwent isolated ACL reconstruction, with or without meniscal procedure, using BPTB or HT autografts between January 2016 and December 2022 were retrospectively reviewed, with follow‐up data collected at a minimum of two years postoperatively, either through clinical records or patient contact.

Patients aged over 50 years with magnetic resonance imaging (MRI) confirmed complete ACL rupture who underwent isolated ACL reconstruction, with or without meniscal procedure, using BPTB or HT autografts at our institution were enroled. Surgical indications included: One or more episodes of knee instability after ACL rupture event, concomitant meniscal tears that need to be repaired, patients who regularly engaged in pivot sports, patients with physically demanding occupations. Additionally, all patients presented with a preoperative Lachman test Grade I or higher. All procedures were performed by the same group of experienced knee surgeons. The choice of graft type was determined by the operating surgeon in consultation with the patient, after discussing the general characteristics of each option.

Exclusion criteria included patients requiring ACL reconstruction as part of multiligament surgeries (classified as Schenck Grade 1–5 [46]), revision ACL reconstruction surgeries, associated lower limb fractures, patients who received allografts or quadriceps tendon autograft, Kellgren–Lawrence Grade 3 or 4 osteoarthritis [27] as assessed preoperatively by the operating surgeon or surgical team using standard radiographs, or patients with insufficient follow‐up.

### Surgical technique

All ACL reconstructions were performed using a standardised anatomic single‐bundle technique. Femoral tunnels were created with an outside‐in approach, and tibial tunnels with an angled tibial guide. Graft fixation was achieved with interference screws on both femoral and tibial sides. BPTB grafts included bone plugs, while hamstring grafts were prepared in a quadrupled or quintupled configuration.

### Postoperative rehabilitation

Postoperative rehabilitation began the day after surgery. For isolated ACL reconstruction, gait retraining started with two crutches, weight‐bearing as tolerated, full range of motion without restrictions, and isometric quadriceps strengthening. In the following months, rehabilitation prioritised crutch discontinuation, stationary bike, range of motion recovery (emphasising extension), proprioception, and strengthening of the hamstrings, quadriceps, and gluteal muscles. Return to sports was permitted at 4 months for non‐pivoting sports, 6 months for pivoting non‐contact sports, and 10–12 months for pivoting contact sports.

There were no differences in rehabilitation protocols between patients reconstructed with BPTB or HT grafts.

If meniscal repair was performed in conjunction with ACL reconstruction, rehabilitation differed compared to isolated ACL reconstructions. However, the specific type of autograft used did not influence the rehabilitation protocol.

For meniscal repairs involving longitudinal tears of the posterior horn, body, or bucket‐handle lesions, rehabilitation started with gait retraining using two crutches and partial weight‐bearing with the knee in full extension. A hinged knee brace was used to allow a controlled range of motion (ROM) from 0° to 90° during the first six to eight postoperative weeks. After this period, the brace was discontinued, and crutch use was gradually reduced.

In contrast, for meniscal repairs involving radial tears or meniscal root reinsertion, non‐weight‐bearing was required for six weeks, followed by a gradual transition to weight‐bearing using crutches. In these cases, a hinged knee brace was also used initially, permitting an ROM of 0°–90°. After the first two months, the rehabilitation of these patients resembled that of patients who had undergone isolated ACL reconstructions.

### Patients evaluation

Demographic characteristics, type of autograft, complications, and postoperative follow‐up outcomes were recorded, including infection, stiffness, failure of ACL reconstruction (defined as a new episode of clinical instability and/or an MRI consistent with graft re‐rupture), or meniscal repair when applicable, and the need for surgical reintervention for other reasons.

The functional outcomes were evaluated between 24 and 30 months postoperatively through phone calls and via email using patient‐reported outcome measures (PROMs) specific to knee injuries. The Abbreviate Quality of Life Knee Osteoarthritis Outcome Score (KOOS QoL), Kujala Score, and the Lysholm scale were utilised to evaluate functionality, patellofemoral symptoms, and disability associated with these injuries.

To further characterise these outcomes, the Lysholm scores were categorised according to standard cutoff points as follows: ‘excellent’ (95–100), ‘good’ (84–94), ‘fair’ (65–83), and ‘poor’ (<65) [51]. Additionally, we assessed the proportion of patients who achieved the Patient Acceptable Symptom State (PASS) for both the Lysholm and KOOS QoL scales. Since no established PASS thresholds are available for ACL reconstruction in patients over 50 years of age, we applied the thresholds proposed by Foissey et al. [17] for the Lysholm scale and by Muller et al. [34] for the KOOS QoL subscale.

A minimum of 2‐year follow‐up period was defined as adequate and safe for diagnosing complications prior to the final evaluation.

Study data were collected and managed using REDCap (Research Electronic Data Capture), a secure, web‐based software platform designed to support data capture for research studies.

### Statistical analysis

Two groups were compared in the present study: patients who underwent ACL reconstruction with a BPTB autograft (Group 1) and those who underwent ACL reconstruction with a hamstring tendon (HT) autograft (Group 2).

Continuous variables were expressed as means ± standard deviation where appropriate, while the dichotomous variables were expressed as the number and percentage of patients. The Shapiro–Wilk normality test was used to assess the normality of distributions.

The independent samples t‐test was used to compare continuous variables between cohorts and for non normal distribution, Mann–Whitney test was performed. Categorical variables were analysed using the chi‐square test. We defined graft type, femoral tunnel technique, meniscal procedures/repairs, and second surgery as variables subject to multivariate analysis and we performed multiple linear regression analysis. A priori power analysis was not performed because all eligible patients were included in the study.

Statistical analysis was performed using STATA (v17; StataCorp 2021, Chicago, IL). A significance level of *p* < 0.05 (95% CI) was set to designate statistical significance. Effect sizes were also calculated to complement the interpretation of statistically significant findings.

## RESULTS

**Figure 1:**
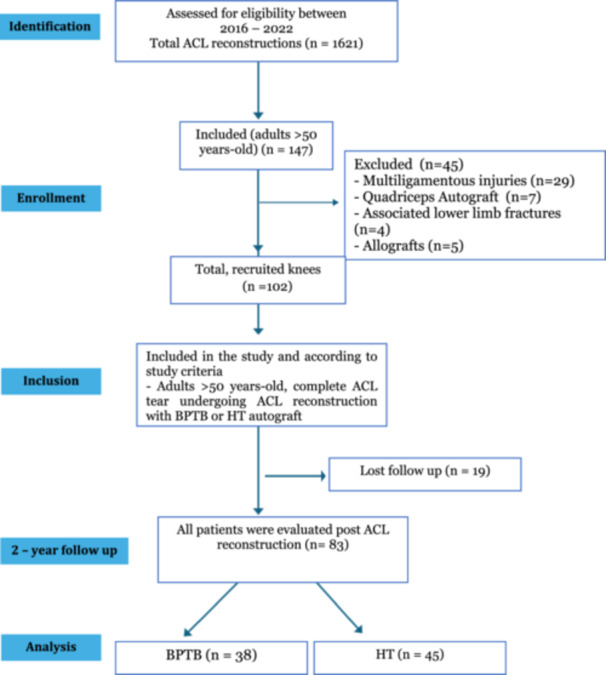
Inclusion criteria for patients enroled in the study. ACL, anterior cruciate ligament; BPTB, bone–patellar tendon–bone; HT, hamstring tendon.

A total of 1621 ACL reconstructions were identified in our database during the evaluated period, of which 147 involved patients over 50 years old. Forty‐five of them were not included in the study due to our exclusion criteria, and 19 were excluded due to loss of follow‐up. Lastly, 83 patients were included in our study as specified in Figure 1.

No lateral extra‐articular procedures (LEAP) were performed.

### Patients characteristics

Eighty three knees in the same number of patients were included of whom 52 were male (62.6%). The mean age was 53.5 years (standard deviation [SD], 3 years; range, 50–63 years), and their average body mass index (BMI) was 27.5 kg/m² (SD 3.4 kg/m^2^). Fourteen patients (8.8%) had type 2 diabetes. In 39 knees (46.9%), ACL reconstruction was performed on the right side. Mean follow‐up was 56.3 months (range [24–107 months]) (Table 1).

**Table 1:** Demographics.

	Total (*n* = 83)	Subgroups		
		HT (*n* = 45)	BPTB (*n* = 38)	*p* value
Age, mean (SD)	53.53 (3.0)	53.6 (2.8)	53.44 (3.3)	0.819
Male, *n* (%)	52 (62.65%)	27 (60%)	25 (65.79%)	0.587
Right, *n* (%)	39 (46.99%)	21 (46.67%)	18 (47.37%)	0.949
Medical history, *n* (%)				
Hypertension	4 (4.82%)	4 (8.9%)	0	0.060
Type 2 diabetes	14 (16.9%)	8 (17.8%)	6 (15.8%)	0.126
Smoker	22 (26.5%)	13 (28.9%)	9 (23.7%)	0.592
BMI, mean (SD)	27.5 (3.5)	27.12 (3.7)	28.0 (3.1)	0.246
Injury mechanism				
Unknown, *n* (%)	2 (2.4%)	2 (4.4%)		
Sports related, *n* (%)	20 (24.1%)	11 (24.4%)	9 (23.7%)	
MVA	9 (10.9%)	5 (11.1%)	4 (10.5%)	
Pivoting, *n* (%)	28 (33.7%)	17 (37.8%)	11 (28.9%)	
Torsion climbing stairs, *n* (%)	7 (8.4%)	2 (4.4%)	5 (13.2%)	
Fall from height, *n* (%)	10 (12.1%)	5 (11.1%)	5 (13.2%)	
Other, *n* (%)	7 (8.4%)	3 (6.7%)	4 (10.5%)	
Kellgren–Lawrence grade				
0	40 (48.8%)	21 (46.7%)	19 (50%)	0.827
I	35 (42.2%)	19 (42.2%)	16 (42.1%)	1.000
II	8 (9.6%)	5 (11.1%)	3 (7.9%)	0.721
Follow up, mean (range)	55.95 (17–107)	68.2 (18–107)	41.44 (17–101)	**<0.001**

*Note*: Age expressed in years, follow‐up expressed in months. Bold figures indicate statistical significance (*p* < 0.05). No cases of Kellgren–Lawrence Grade III or IV were identified.

Abbreviations: BMI, body mass index; BPTB, bone–patellar tendon–bone; HT, hamstring tendon; MVA, motor vehicle accident; SD, standard deviation; yo, years old.

The most frequently described injury mechanism was pivoting with or without a ground level fall in 28 cases (33.7%) followed by sports related injuries in 20 knees (24.1%).

Mean graft diameter (based on tunnel size) was 8.7 mm (SD 0.96), with 8.0 mm (SD 0.6) in HT and 9.4 mm (SD 0.81) in BPTB (*p* < 0.001). Meniscal procedures were performed in 60 cases (72.3%; *p* 0.794). Among them, 19 patients (22.9%; *p* = 0.714) underwent meniscal repair, accounting for a total of 20 repaired meniscus, 12 in the HT group and 8 in the BPTB group, as one patient had both the medial and lateral meniscus repaired. In addition, 46 patients (55.4%; *p* = 0.390) underwent partial meniscectomy (Table 2).

**Table 2:** Surgical characteristics.

	Total	Subgroups		
		HT	BPTB	*p* value
Diameter (mm), mean (SD)	8.6 (0.9)	8 (0.6)	9.3 (0.8)	**<0.001**
Meniscal injuries, *n* (%)	19 (22.9%)	10 (22.2%)	9 (23.7%)	0.794
Meniscal procedures, *n* (%)	60 (72.29%)	32 (71.1%)	28 (73.7%)	0.794
Meniscectomies, *n* (%)	46 (55.42%)	23 (51.1%)	23 (60.5%)	0.390
Medial meniscectomy, *n* (%)	36 (43.37%)	17 (37.8%)	19 (50%)	0.263
Lateral meniscectomy, *n* (%)	23 (27.71%)	12 (26.7%)	11 (28.9%)	0.817
Meniscal repairs, *n* (%)	20 (24.1%)	12 (26.6%)	8 (21.9%)	0.714
Medial repair, *n* (%)	14 (16.87%)	11 (24.4%)	3 (7.9%)	**0.045**
Lateral repair, *n* (%)	6 (7.23%)	1 (2.2%)	5 (13.2%)	0.055
Type of meniscal repairs				0.228
Vertical, *n* (%)	7 (8.4%)	5 (11.1%)	2 (5.3%)	
Bucket handle, *n* (%)	5 (6.0%)	4 (8.8%)	1 (2.6%)	
Radial, *n* (%)	2 (2.4%)	0	2 (5.3%)	
Horizontal, *n* (%)	2 (2.4%)	1 (2.22%)	1 (2.6%)	
Root, *n* (%)	2 (2.4%)	0	2 (5.3%)	
Oblique, *n* (%)	1 (1.2%)	1 (2.2%)	0	
Ramp, *n* (%)	1 (1.2%)	1 (2.2%)	0	
Location				0.510
Posterior horn, *n* (%)	14 (16.9%)	8 (17.8%)	5 (13.2%)	
Whole meniscus, *n* (%)	6 (7.2%)	6 (6.4%)	3 (7.9%)	
Repaired tear size (mm), mean (SD)	23.1 (16.2)	23.2 (14.4)	24.5 (19.1)	0.862

*Note*: Diameter and size expressed in mm (milimeters). Bold figures indicate statistical significance (*p* < 0.05).

Abbreviations: BPTB, bone–patellar tendon–bone; HT, hamstring tendon; SD, standard deviation.

### PROMs

Regarding PROMs, there was a mean Lysholm score of 84.5 (SD 18.75; *p* = 0.934), a mean Kujala score of 83.3 (SD 18.66; *p* = 0.901), and a mean KOOS QoL score of 68.9 (SD 26.04; *p* = 0.649). No significant differences were found between the two groups (Table 3).

**Table 3:** PROMs.

	Overall	Subgroups		
		HT	BPTB	*p* value
Lysholm, mean (SD)	84.5 (18.75)	83.1 (21.85)	86.1 (14.36)	0.934
Kujala, mean (SD)	83.3 (18.66)	82.2 (20.49)	84.5 (16.43)	0.901
KOOS QoL mean (SD)	68.9 (26.04)	69.0 (23.44)	68.7 (28.32)	0.649

Abbreviations: BPTB, bone–patellar tendon–bone; HT, hamstring tendons; KOOS, Knee injury and Osteoarthritis Outcome Score; PROMs, patient‐reported outcome measures; QoL, quality of life; SD, standard deviation.

Based on the predefined cutoffs, the distribution of Lysholm score categories (‘excellent’, ‘good’, ‘fair’, and ‘poor’) was similar between groups. Likewise, no significant differences were observed in the proportion of patients achieving the PASS threshold for either the Lysholm or KOOS QoL scales (Table 4).

**Table 4:** Categorised PROMs.

	Overall	Subgroups		
		HT	BPTB	*p* value
Lysholm achieving PASS (>86)				0.562
Yes	53 (63.9%)	30 (66.7%)	23 (60.5%)	
No	30 (36.1%)	15 (33.3%)	15 (39.5%)	
Lysholm categorised				0.145
Excellent (95–100)	32 (38.6%)	19 (42.2%)	13 (34.2%)	
Good (84–94)	26 (31.3%)	15 (33.3%)	11 (28.9%)	
Fair (65–83)	10 (12.1%)	2 (4.4%)	8 (21.1%)	
Poor (<65)	15 (18.1%)	9 (20%)	6 (15.7%)	
KOOS QoL achieving PASS (>62.5)				0.555
Yes	54 (65.1%)	28 (62.2%)	26 (68.4%)	
No	29 (34.9%)	17 (37.8%)	12 (31.6%)	

Abbreviations: BPTB, bone–patellar tendon–bone; HT, hamstring tendons; KOOS, Knee injury and Osteoarthritis Outcome Score; PASS, Patient Acceptable Symptom State; PROMs, patient‐reported outcome measures; QoL, quality of life.

A multivariate linear regression model was fitted to determine the association between graft type, meniscal repairs, second surgeries and PROMs. The model showed a statistically significant association with Lysholm score (*p* = 0.048) and KOOS QoL (*p* = 0.040), but failed to demonstrate a significant association with Kujala score (*p* = 0.098). When observing each independent variable, only second surgeries had a significant association with a decrease in all PROMs: with each second surgery, Lysholm score decreased by 17.4 (*p* = 0.003), Kujala score decreased by 15.9 (*p* = 0.007) and KOOS QoL decreased by 25.3 (*p* = 0.002). In the case of Kujala score, this means that the whole model does not explain the variations in PROMs, but only second surgeries as an isolated factor is the most meaningful in the variation of PROMs (Table 5).

**Table 5:** Multivariate analysis.

	Variables				
	Graft type	Graft diameter	Meniscal repair	Second surgery	*p* value
PROM					
Lysholm, coefficient (CI); *p*‐value	−2.582 (−13.895 to 8.729); 0.651	1.486 (−4.180 to 7.153); 0.603	4.158 (−5.486 to 13.803); 0.393	−17.397 (−28.714 to −6.081); **0.003**	**0.048**
Kujala, coefficient (CI); *p*‐value	−2.247 (−13.634 to 9.139); 0.695	0.910 (−4.794 to 6.614); 0.752	3.581 (−6.127 to 13.289); 0.465	−15.957 (−27.348 to −4.566); **0.007**	0.098
KOOS QoL, coefficient (CI); *p*‐value	−7.347 (−23.017 to 8.321); 0.353	1.445 (−6.404 to 9.294); 0.715	7.806 (−5.553 to 21.165); 0.248	−25.273 (−40.948 to 9.597); **0.002**	**0.040**

*Note*: Bold figures indicate statistical significance (*p* < 0.05).

Abbreviations: CI, confidence interval; KOOS, Knee injury and Osteoarthritis Outcome Score; PROMs, patient‐reported outcome measures; QoL, quality of life.

### Reoperations and complications

When analysing the need for second surgeries, 14 cases (16.87%) underwent a second procedure, with an estimated odds ratio (OR) for the need of a second surgery of 6.54 (1.36–31.45; *p* = 0.019) with the use of HT graft compared with BPTB. The most frequent reinterventions were cyclops lesion resection, performed in four cases, with three cases (6.6%) in HT grafts and one case (2.6%) in BPTB grafts (*p* = 0.392), and meniscal reintervention, also in four cases. The latter included three patients who had previously undergone medial meniscal repair and one patient who had undergone partial meniscectomy during the initial surgery, with all four cases (8.8%) occurring in HT grafts (*p* = 0.060). We observed that among the 17 meniscal repairs that did not fail, 12 cases were confirmed by MRI performed between 8 and 34 months postoperatively, demonstrating proper meniscal healing, while in the remaining five cases, the absence of failure was assessed clinically based on the lack of meniscal symptoms. In the case of failed meniscal repairs, all three instances involved medial meniscus tears—one was a longitudinal vertical tear of the posterior horn, and the other two were bucket‐handle tears. In all cases, re‐tear was confirmed by MRI. The first case involved a 58‐year‐old male patient who underwent partial meniscectomy eight months after the initial repair due to persistent medial pain, despite the absence of joint locking. The second case was a 62‐year‐old female patient with a bucket‐handle tear repaired using combined inside‐out and all‐inside sutures, who experienced failure due to joint locking 10 months postoperatively. The third case was a 51‐year‐old female patient, also with a repaired bucket‐handle tear, who required reintervention at 42 months due to persistent posteromedial pain despite physiotherapy. There was no joint locking, and MRI revealed a complex re‐tear of the posterior horn of the medial meniscus.

Another patient in the HT group developed a condition consistent with septic arthritis following a new traumatic event in the operated knee at 20 months of follow‐up. The case was successfully managed with surgical debridement and intravenous antibiotic therapy for four weeks. MRI and arthroscopic evaluation confirmed graft continuity. Additionally, two patients required the removal of the interference screw due to associated discomfort, one in the femur and the other in the tibial fixation. There were no reinterventions for ACL graft revision. One patient in the HT reconstruction group sustained a partial graft rupture, confirmed by MRI following a new traumatic event. However, due to the absence of clinical failure, conservative management was chosen (Table 6). After analysing each complication and reintervention, only septic arthritis (*p* = 0.355), arthroscopic lysis of adhesions (*p* = 0.659), and cyclops lesion (*p* = 0.392) could be considered directly related to the type of graft used, and none of them showed a statistically significant difference between graft types.

**Table 6:** Complications.

	Total	Subgroups		
		HT	BPTB	*p* value
Second surgery, *n* (%)	14 (16.9%)	12 (26.6%)	2 (5.2%)	**0.009**
Septic arthritis, *n* (%)	1 (1.2%)	1 (2.2%)	0	0.355
Arthroscopic lysis of adhesions, *n* (%)	3 (3.6%)	2 (4.4%)	1 (2.6%)	0.659
Cyclops, *n* (%)	4 (4.8%)	3 (6.6%)	1 (2.6%)	0.392
Meniscal procedure, *n* (%)	4 (4.8%)	4 (8.8%)	0	0.060
Meniscal repair meniscectomy, *n* (%)	3 (3.6%)	3 (6.6%)	0	0.105
Second meniscectomy, *n* (%)	1 (1.2%)	1 (2.2%)	0	0.355
Femoral screw removal, *n* (%)	1 (1.2%)	1 (2.2%)	0	0.355
Tibial screw removal, *n* (%)	1 (1.2%)	1 (2.2%)	0	0.355

*Note*: Bold figure indicate statistical significance (*p* < 0.05).

Abbreviations: BPTB, bone–patellar tendon–bone; HT, hamstring tendons.

## DISCUSSION

The most important finding of this study was that patients over 50 years old who underwent ACL reconstruction with BPTB or HT autografts achieved good outcomes and functional scores regardless of the type of autograft used. Secondly, although the overall reoperation rate was higher in the HT group, no statistically significant differences were observed in reinterventions directly attributable to the type of graft. Additionally, patients requiring surgical reintervention demonstrate worse long‐term functional outcomes compared to those who do not. Lastly, patients undergoing concomitant meniscal repair with ACL reconstruction exhibit a low failure rate, which is consistent with previous studies in younger populations.

The increase in life expectancy among the older population has led to a rise in ACL injuries and reconstructions in older patients [5, 45]. The outcomes of ACL reconstruction in this age group have been progressively documented, showing good functional results and low failure rates in patients over 40 years old [29, 31, 42, 43, 52, 58], as well as in those over 50 years old [2, 10, 12, 15, 16, 33, 37, 51, 57], with outcomes comparable to those in patients under 50 [53], under 40 [22], and even under 30 years old [9, 38].

The two most used autografts for ACL reconstruction remain HT, followed by BPTB [7, 19, 56]. In this regard, literature comparing these graft types in patients over 50 years old remains scarce. In their comparative study of 55 patients, Bali et al. [2] reported good functional outcomes at one‐year follow‐up, with mean International Knee Documentation Committee (IKDC) and Lysholm scores of 73.6 and 89.7, respectively, without significant differences between techniques. Similarly, in their prospective study on female patients, Kautzner et al. [26] found mean Lysholm scores of 88 for BPTB and 90 for HT, also without significant differences. These findings align with those observed in our patients, where we obtained mean Lysholm scores of 86.1 for BPTB and 83.1 for HT in evaluations conducted between 24 and 30 months of follow‐up.

Additionally, we chose to evaluate the QoL subscale of the KOOS, which has been shown to have a high response rate [1] in patients undergoing ACL reconstruction. Our results were slightly lower but consistent with those previously reported in the literature, with a mean score of 68.9. Ogunleye et al. [37] reported a mean score of 73.3 in their study on patients over 55 years old using HT autografts, while Micicoi et al. [33], in their study with BPTB autografts and a minimum follow‐up of 10 years, reported a mean KOOS QoL score of 71.5.

Regarding patellofemoral symptoms and anterior knee pain, the literature remains inconclusive as to whether BPTB leads to increased symptoms in this regard. Some studies report higher patellofemoral symptomatology [32, 40, 54], while others do not find significant differences [24, 48]. However, none specifically focus on the patient population of our study. In our cohort, we did not find significant differences in Kujala scores, with both groups achieving acceptable outcomes.

Concerning complications, we observed a 16.9% rate of surgical reinterventions during follow‐up, with the most frequent being cyclops lesion resections (four cases) and meniscectomies (also four cases). Of the latter, three occurred in patients who had undergone meniscal repair during the initial surgery. The third most common reintervention was for arthrofibrosis (three cases). Although the overall rate of reinterventions was higher in the HT group, no statistically significant differences were found when analysing reinterventions directly related to the type of graft.

With respect to cyclops lesions, we did not find studies in similar populations reporting this complication. However, extrapolating to the general population, Noailles [36] identified an incidence of up to 10.9%, with preoperative range of motion restriction, a narrow intercondylar notch, and an excessively anterior tibial tunnel position as potential risk factor. For arthrofibrosis, reported rates range from 0% to 12% [9, 53].

Regarding meniscal repairs, we observed a failure rate of 15% (three out of 20 repairs). In patients over 50 years old undergoing meniscal repair, both Cinque et al. [9] and Kim et al. [28] reported generally good outcomes in 16 and 21 meniscal repairs performed alongside ACL reconstruction in comparative studies between patients over and under 50 years old, although they did not specifically focus on meniscal repair failure rates.

The reported failure rate of meniscal repairs in the general population is approximately 14.8% [11, 47], while in patients over 40 years old, it is 15.5% [23], reaching 22.2% in patients over 60 [21]. In this context, we consider a 15% failure rate to be reasonable.

It is important to highlight that reinterventions of any kind had a significant impact on the PROMs of the evaluated subjects, reducing scores by up to 15.7 points in the case of the Kujala score and up to 24.9 points for the KOOS QoL. Therefore, identifying the causes and risk factors for reoperations is crucial to minimising their occurrence.

This study is not without limitations, primarily due to its retrospective nature, which includes the lack of randomisation, potentially leading to selection bias. Additionally, the absence of preoperative evaluations and scores prevents a baseline comparison between both groups and an assessment of their potential improvement over time. Moreover, key variables related to the therapeutic goal of ACL reconstruction, such as return to sports or work, were not evaluated. Additionally, no objective stability tests were performed, which could have provided further insight into the outcomes of both techniques. Furthermore, the average follow‐up is limited, and we lack standardised radiological evaluations over an extended period to identify important outcomes, such as the long‐term development of patellofemoral osteoarthritis, which could be significant in the case of choosing BPTB as a graft [30, 33]. Furthermore, we lack standardised long‐term radiological evaluations that could have helped identify important outcomes such as the development of osteoarthritis.

## CONCLUSION

In patients over 50 years old, ACL reconstruction with either BPTB or HT autografts is a safe procedure that yields good functional outcomes, with no significant differences between graft types. The overall complication rate is comparable to that of younger populations, suggesting that age alone should not be considered a contraindication for ACL surgery.

## AUTHOR CONTRIBUTIONS

Rodrigo Olivieri designed the study, coordinated the team, participated in data collection, abstract writing, manuscript drafting, and bibliographic references, and is the corresponding author. José I. Laso was responsible for synthesis and writing of results, statistical analysis, and tables preparation. Carlos Rojas participated in data collection and development for approval by the ethics committee of our institution. Nicolás Franulic participated in data collection and worked on abstract writing and methods. Felipe Bustos and Roberto Oyarce participated in data collection. Nicolás Gaggero worked on abstract writing and discussion. Alberto Grassi participated in advising the statistical analysis and in correcting the drafting of the introduction, methods, and discussion sections of the manuscript.

## CONFLICT OF INTEREST STATEMENT

The author, Rodrigo Olivieri, reports speaking fees from Johnson & Johnson Medtech. The author, Nicolás Gaggero, reports speaking fees from Johnson & Johnson Medtech, and Smith+Nephew. The remaining authors declare no conflicts of interest.

## ETHICS STATEMENT

The study was conducted in accordance with the ethical standards outlined in the Declaration of Helsinki and Resolution 008430 of 1993. It was approved by the Institutional Ethics Committee (internal code CEC/02/2025), and informed consent was obtained from all patients participating in the study at the time the functional scales were administered. Informed consent was obtained from all individual participants included in the study.

## Data Availability

The data that support the findings of this study are available on request from the corresponding author. The data are not publicly available due to privacy or ethical restrictions.

## References

[1] Abed V , Kapp S , Nichols M , Castle JP , Landy DC , Conley C , et al. Lysholm and KOOS QoL demonstrate high responsiveness in patients undergoing anterior cruciate ligament reconstruction: a systematic review and meta‐analysis of randomized clinical trials. Am J Sports Med. 2024;52:3161–3166. 38352999 10.1177/03635465231219966

[2] Bali T , Nagraj R , Kumar MN , Chandy T . Outcomes of the patellar tendon and hamstring graft anterior cruciate ligament reconstructions in patients aged above 50 years. Indian J Orthop. 2015;49:615–619. 26806968 10.4103/0019-5413.168760 PMC4705727

[3] Banovetz MT , Kennedy NI , LaPrade RF , Engebretsen L , Moatshe G . Biomechanical considerations for graft choice in anterior cruciate ligament reconstruction. Annals Joint. 2023;8:17. 10.21037/aoj-22-50 PMC10929340 38529237

[4] Best MJ , Zikria BA , Wilckens JH . Anterior cruciate ligament injuries in the older athlete. Sports Health. 2021;13:285–289. 33301359 10.1177/1941738120953426 PMC8083147

[5] Buller LT , Best MJ , Baraga MG , Kaplan LD . Trends in anterior cruciate ligament reconstruction in the United States. Orthop J Sports Med. 2015;3:2325967114563664. 26535368 10.1177/2325967114563664 PMC4555588

[6] Calvert ND , Smith A , Ackland T , Kuster MS , Ebert J . Kneeling difficulty is common following anterior cruciate ligament reconstruction with hamstring autograft and correlates with outcome measures. Arch Orthop Trauma Surg. 2020;140:913–921. 32128629 10.1007/s00402-020-03401-x

[7] Cerciello S , Ollivier M , Kocaoglu B , Khakha RS , Seil R . ACL surgical trends evolve in the last five years for young European surgeons: results of the survey among the U45 ESSKA members. Knee Surg Sports Traumatol Arthrosc. ESSKA U45 Committee 2023;31:619–625. 35699743 10.1007/s00167-022-07005-3

[8] Ciccotti MG , Lombardo SJ , Nonweiler B , Pink M . Non‐operative treatment of ruptures of the anterior cruciate ligament in middle‐aged patients: results after long‐term follow‐up. J Bone Joint Surg. 1994;76:1315–1321. 8077261 10.2106/00004623-199409000-00006

[9] Cinque ME , Chahla J , Moatshe G , DePhillipo NN , Kennedy NI , Godin JA , et al. Outcomes and complication rates after primary anterior cruciate ligament reconstruction are similar in younger and older patients. Orthop J Sports Med. 2017;5:2325967117729659. 29051896 10.1177/2325967117729659 PMC5637972

[10] Costa GG , Grassi A , Perelli S , Agrò G , Bozzi F , Lo Presti M , et al. Age over 50 years is not a contraindication for anterior cruciate ligament reconstruction. Knee Surg Sports Traumatol Arthrosc. 2019;27:3679–3691. 30944945 10.1007/s00167-019-05450-1

[11] Costa GG , Grassi A , Zocco G , Graceffa A , Lauria M , Fanzone G , et al. What is the failure rate after arthroscopic repair of bucket‐handle meniscal tears? A systematic review and meta‐analysis. Am J Sports Med. 2022;50:1742–1752. 34161741 10.1177/03635465211015425

[12] Dahm DL , Wulf CA , Dajani KA , Dobbs RE , Levy BA , Stuart MA . Reconstruction of the anterior cruciate ligament in patients over 50 years. J Bone Joint Surg Br. 2008;90–B:1446–1450. 10.1302/0301-620X.90B11.21210 18978263

[13] Dhammi IK , Rehan‐Ul‐Haq H , Kumar S . Graft choices for anterior cruciate ligament reconstruction. Indian J Orthop. 2015;49:127–128. 26015598 10.4103/0019-5413.152393 PMC4436475

[14] Ehlinger M , Panisset JC , Dejour D , Gonzalez JF , Paihle R , Favreau H , et al. Anterior cruciate ligament reconstruction in the over‐50s: a prospective comparative study between surgical and functional treatment. Orthop Traumatol: Surg Res. Francophone Arthroscopy Society (SFA) 2021;107:103039. 34375770 10.1016/j.otsr.2021.103039

[15] Fayard JM , Wein F , Ollivier M , Paihle R , Ehlinger M , Lustig S , et al. Factors affecting outcome of ACL reconstruction in over‐50‐year‐olds. Orthop Traumatol: Surg Res. French Arthroscopic Society 2019;105:S247–S251. 31564634 10.1016/j.otsr.2019.09.011

[16] Figueroa D , Figueroa F , Calvo R , Vaisman A , Espinoza G , Gili F . Anterior cruciate ligament reconstruction in patients over 50 years of age. Knee. 2014;21:1166–1168. 25174853 10.1016/j.knee.2014.08.003

[17] Foissey C , Thaunat M , Caron E , Haidar I , Vieira TD , Gomes L , et al. Combining anterior cruciate ligament reconstruction with lateral extra‐articular procedures in skeletally immature patients is safe and associated with a low failure rate. Arthrosc Sports Med Rehabil. 2022;4:e1941–e1951. 36579042 10.1016/j.asmr.2022.08.002 PMC9791843

[18] Freedman KB . Editorial commentary: graft choice for anterior cruciate ligament reconstruction: will there ever be a correct answer? Probably not. Arthroscopy. 2020;36:1647–1648. 32503775 10.1016/j.arthro.2020.03.005

[19] Grassi A , Carulli C , Innocenti M , Mosca M , Zaffagnini S , Bait C . New trends in anterior cruciate ligament reconstruction: a systematic review of national surveys of the last 5 years. Joints. SIGASCOT Arthroscopy Committee 2018;06:177–187. 10.1055/s-0038-1672157 PMC6301855 30582107

[20] Grevnerts HT , Sonesson S , Gauffin H , Ardern CL , Stålman A , Kvist J . Decision making for treatment after ACL injury from an orthopaedic surgeon and patient perspective: results from the NACOX study. Orthop J Sports Med. 2021;9:23259671211005090. 33948447 10.1177/23259671211005090 PMC8053763

[21] Husen M , Kennedy NI , Till S , Reinholz A , Stuart MJ , Krych AJ , et al. Benefits of meniscal repair in selected patients aged 60 years and older. Orthop J Sports Med. 2022;10:23259671221117491. 36081411 10.1177/23259671221117491 PMC9445464

[22] Iorio R , Iannotti F , Ponzo A , Proietti L , Redler A , Conteduca F , et al. Anterior cruciate ligament reconstruction in patients older than fifty years: a comparison with a younger age group. Int Orthop. 2018;42:1043–1049. 29532113 10.1007/s00264-018-3860-8

[23] Jaibaji R , Khaleel F , Jaibaji M , Volpin A . Outcomes of meniscal repair in patients aged 40 and above: a systematic review. J Clin Med. 2023;12:6922. 37959387 10.3390/jcm12216922 PMC10649032

[24] Jansson KA , Linko E , Sandelin J , Harilainen A . A prospective randomized study of patellar versus hamstring tendon autografts for anterior cruciate ligament reconstruction. Am J Sports Med. 2003;31:12–18. 12531751 10.1177/03635465030310010501

[25] Jokl P , Kaplan N , Stovell P , Keggi K . Non‐operative treatment of severe injuries to the medial and anterior cruciate ligaments of the knee. J Bone Joint Surg. 1984;66:741–744. 6725321

[26] Kautzner J , Kos P , Hanus M , Trc T , Havlas V . A comparison of ACL reconstruction using patellar tendon versus hamstring autograft in female patients: a prospective randomised study. Int Orthop. 2015;39:125–130. 25128968 10.1007/s00264-014-2495-7

[27] Kellgren JH , Lawrence JS . Radiological assessment of osteo‐arthrosis. Ann Rheum Dis. 1957;16:494–502. 13498604 10.1136/ard.16.4.494 PMC1006995

[28] Kim DK , Park G , Kuo LT , Park WH . Patients older than 50 years had similar results of knee strength and anteroposterior stability after ACL reconstruction compared to younger patients. Knee Surg Sports Traumatol Arthrosc. 2019;27:230–238. 30600340 10.1007/s00167-018-5342-3

[29] Legnani C , Terzaghi C , Borgo E , Ventura A . Management of anterior cruciate ligament rupture in patients aged 40 years and older. J Orthop Traumatol. 2011;12:177–184. 22075673 10.1007/s10195-011-0167-6 PMC3225626

[30] Lucidi GA , Agostinone P , Di Paolo S , Dal Fabbro G , Serra M , Viotto M , et al. Long‐term outcomes after anterior cruciate ligament reconstruction with 3 different surgical techniques: a prospective randomized clinical and radiographic evaluation at a minimum of 20 years' follow‐up. Orthop J Sports Med. 2025;13:23259671241302348. 39886263 10.1177/23259671241302348 PMC11780662

[31] Mall NA , Frank RM , Saltzman BM , Cole BJ , Bach Jr. BR . Results after anterior cruciate ligament reconstruction in patients older than 40 years: how do they compare with younger patients? A systematic review and comparison with younger populations. Sports Health. 2016;8:177–181. 26674619 10.1177/1941738115622138 PMC4789931

[32] Marques FS , Barbosa PHB , Alves PR , Zelada S , Nunes RPS , de Souza MR , et al. Anterior knee pain after anterior cruciate ligament reconstruction. Orthop J Sports Med. 2020;8(10):2325967120961082. 33195725 10.1177/2325967120961082 PMC7605008

[33] Micicoi G , Fairag R , Machado A , Douiri A , Bronsard N , Ernat J , et al. Anterior cruciate ligament reconstruction in patients older than 50 years: a descriptive study with minimum 10‐year follow‐up. Orthop J Sports Med. 2024;12:23259671241292071. 39628765 10.1177/23259671241292071 PMC11610015

[34] Muller B , Yabroudi MA , Lynch A , Lai CL , van Dijk CN , Fu FH , et al. Defining thresholds for the patient acceptable symptom state for the IKDC subjective knee form and KOOS for patients who underwent ACL reconstruction. Am J Sports Med. 2016;44:2820–2826. 27474383 10.1177/0363546516652888

[35] Musahl V , Engler ID , Nazzal EM , Dalton JF , Lucidi GA , Hughes JD , et al. Current trends in the anterior cruciate ligament part II: evaluation, surgical technique, prevention, and rehabilitation. Knee Surg Sports Traumatol Arthrosc. 2022;30:34–51. 34865182 10.1007/s00167-021-06825-z

[36] Noailles T , Chalopin A , Boissard M , Lopes R , Bouguennec N , Hardy A . Incidence and risk factors for cyclops syndrome after anterior cruciate ligament reconstruction: a systematic literature review. Orthop Traumatol: Surg Res. 2019;105:1401–1405. 31405748 10.1016/j.otsr.2019.07.007

[37] Ogunleye P , Jäger H , Zimmermann F , Balcarek P , Sobau C , Ellermann A , et al. Patients older than 55 years regain sporting and recreational activities after arthroscopic anterior cruciate ligament reconstruction. Knee Surg Sports Traumatol Arthrosc. 2023;31:632–640. 35988115 10.1007/s00167-022-07116-x

[38] Osti L , Papalia R , Del Buono A , Leonardi F , Denaro V , Maffulli N . Surgery for ACL deficiency in patients over 50. Knee Surg Sports Traumatol Arthrosc. 2011;19:412–417. 20734026 10.1007/s00167-010-1242-x

[39] Ostojic M , Indelli PF , Lovrekovic B , Volcarenghi J , Juric D , Hakam HT , et al. Graft selection in anterior cruciate ligament reconstruction: a comprehensive review of current trends. Medicina. 2024;60:2090. 39768969 10.3390/medicina60122090 PMC11678177

[40] Peebles LA , Akamefula RA , Aman ZS , Verma A , Scillia AJ , Mulcahey MK , et al. Following anterior cruciate ligament reconstruction with bone‐patellar tendon‐bone autograft, the incidence of anterior knee pain ranges from 5.4% to 48.4% and the incidence of kneeling pain ranges from 4.0% to 75.6%: a systematic review of level I studies. Arthrosc Sports Med Rehabil. 2024;6:100902. 38562662 10.1016/j.asmr.2024.100902 PMC10982565

[41] Phatama KY , Mustamsir E , Jaya AO , Pradana AS , Putra DP , Hidayat M . Patellofemoral functional outcome of gracilis sparing compared to gracilis sacrificing ACL reconstruction. Annals Med Surg. 2022;84:104940. 10.1016/j.amsu.2022.104940 PMC9732112 36504706

[42] Puzzitiello RN , Sylvia SM , Perrone GS , Bragg JT , Richmond JC , Salzler MJ . Preoperative factors associated with failure to reach the patient acceptable symptom state after anterior cruciate ligament reconstruction in patients aged 40 and older. Knee Surg Sports Traumatol Arthrosc. 2023;31:3204–3211. 36811656 10.1007/s00167-023-07334-x

[43] Roberts J , Puzzitiello R , Salzler M . Anterior cruciate ligament reconstruction in patients over 40 years old shows low failure rates: a systematic review. Arthrosc Sports Med Rehabil. 2024;6:100899. 38706974 10.1016/j.asmr.2024.100899 PMC11065657

[44] Rugg CM , Tucker LY , Ding DY . Nonoperative treatment of anterior cruciate ligament tears with 5‐year follow‐up. Orthop J Sports Med. 2025;13:23259671251314441. 40052181 10.1177/23259671251314441 PMC11881924

[45] Sanders TL , Maradit Kremers H , Bryan AJ , Larson DR , Dahm DL , Levy BA , et al. Incidence of anterior cruciate ligament tears and reconstruction: a 21‐year population‐based study. Am J Sports Med. 2016;44:1502–1507. 26920430 10.1177/0363546516629944

[46] Schenck RC . The dislocated knee. Instr Course Lect. 1994;43:127–136. 9097143

[47] Schweizer C , Hanreich C , Tscholl PM , Blatter S , Windhager R , Waldstein W . Meniscal repair outcome in 3829 patients with a minimum follow‐up from 2 years up to 5 years: a meta‐analysis on the overall failure rate and factors influencing failure. Am J Sports Med. 2024;52:822–831. 37022676 10.1177/03635465231158385

[48] Sgardelis P , Naqvi G , Servant C . Minimally invasive bone‐patellar tendon‐bone graft harvest is associated with less frequent anterior knee pain at rest than hamstring graft harvest after anterior cruciate ligament reconstruction at the 1‐year follow‐up. Arthrosc Sports Med Rehabil. 2023;5:100766. 37529626 10.1016/j.asmr.2023.100766 PMC10387569

[49] Sim K , Rahardja R , Zhu M , Young SW . Optimal graft choice in athletic patients with anterior cruciate ligament injuries: review and clinical insights. Open Access J Sports Med. 2022;13:55–67. 35800660 10.2147/OAJSM.S340702 PMC9255990

[50] Strehl A , Eggli S . The value of conservative treatment in ruptures of the anterior cruciate ligament (ACL). J Trauma: Injury Infect Crit Care. 2007;62:1159–1162. 10.1097/TA.0b013e31805006e7 17495718

[51] Sylvia SM , Gill TJ , Engler ID , Carroll KM , Salzler MJ . Anterior cruciate ligament reconstruction using bone‐patellar tendon‐bone allograft in patients aged 50 and older leads to improved activity levels and acceptable patient‐reported outcomes. Arthrosc Sports Med Rehabil. 2021;3:e1961–e1965. 34977654 10.1016/j.asmr.2021.09.018 PMC8689261

[52] Sylvia SM , Perrone GS , Stone JA , Miltenberg B , Nezwek TA , Zhang Y , et al. The majority of patients aged 40 and older having allograft anterior cruciate ligament reconstruction achieve a patient acceptable symptomatic state. Arthroscopy. 2022;38:1537–1543. 34601008 10.1016/j.arthro.2021.09.024

[53] Tan CW , Hsu WH , Yu PA , Chen CL , Kuo LT , Chi CC , et al. Anterior cruciate ligament reconstruction in patients older than 50 years: a systematic review and meta‐analysis. Orthop J Sports Med. 2020;8:2325967120915698. 32426406 10.1177/2325967120915698 PMC7218932

[54] Tan SHS , Lau BPH , Krishna L . Outcomes of anterior cruciate ligament reconstruction in females using patellar‐tendon‐bone versus hamstring autografts: a systematic review and meta‐analysis. J Knee Surg. 2019;32:770–787. 30212919 10.1055/s-0038-1669916

[55] Thaunat M , Fayard JM , Sonnery‐Cottet B . Hamstring tendons or bone‐patellar tendon‐bone graft for anterior cruciate ligament reconstruction? Orthop Traumatol: Surg Res. 2019;105:S89–S94. 30130660 10.1016/j.otsr.2018.05.014

[56] Tuca M , Valderrama I , Eriksson K , Tapasvi S . Current trends in anterior cruciate ligament surgery: a worldwide benchmark study. J ISAKOS. 2023;8:2–10. 36154898 10.1016/j.jisako.2022.08.009

[57] Weng CJ , Yeh WL , Hsu KY , Chiu C , Chang SS , Chen ACY , et al. Clinical and functional outcomes of anterior cruciate ligament reconstruction with autologous hamstring tendon in patients aged 50 years or older. Arthroscopy. 2020;36:558–562. 31901387 10.1016/j.arthro.2019.08.047

[58] Wierer G , Herbst E , Hoser C , Gföller P , Fink C . High rate of return to activity after ACL reconstruction in patients over 40 years of age: a systematic review. J ISAKOS. 2017;2:200–204.

[59] Wolfson TS , Epstein DM , Day MS , Joshi BB , McGee A , Strauss EJ , et al. Outcomes of anterior cruciate ligament reconstruction in patients older than 50 years of age. Bull Hosp Joint Dis. 2014;72:277–283. 25986352

